# Developmental Trajectories in Primary Schoolchildren Using n-Back Task

**DOI:** 10.3389/fpsyg.2016.00716

**Published:** 2016-05-13

**Authors:** Mónica López-Vicente, Joan Forns, Elisabet Suades-González, Mikel Esnaola, Raquel García-Esteban, Mar Álvarez-Pedrerol, Jordi Júlvez, Miguel Burgaleta, Núria Sebastián-Gallés, Jordi Sunyer

**Affiliations:** ^1^ISGlobal, Centre for Research in Environmental EpidemiologyBarcelona, Spain; ^2^Department of Experimental and Health Sciences, Universitat Pompeu FabraBarcelona, Spain; ^3^CIBER Epidemiología y Salud PúblicaBarcelona, Spain; ^4^Hospital del Mar Medical Research InstituteBarcelona, Spain; ^5^Department of Genes and Environment, Division of Epidemiology, Norwegian Institute of Public HealthOslo, Norway; ^6^Learning Disabilities Unit (UTAE), Neuropediatrics Department, Hospital Sant Joan de Déu, Universitat de BarcelonaBarcelona, Spain; ^7^Environmental Health Department, Harvard T.H. Chan School of Public HealthBoston, MA, USA; ^8^Center for Brain and Cognition, Department of Technology, Universitat Pompeu FabraBarcelona, Spain

**Keywords:** n-back, working memory, neurodevelopment, schoolchildren, follow-up, population study, longitudinal study

## Abstract

**Background:** Neuropsychological instruments to assess cognitive trajectories during childhood in epidemiological studies are needed. This would improve neurodevelopment characterization in order to identify its potential determinants. We aimed to study whether repeated measures of n-back, a working memory task, detect developmental trajectories in schoolchildren during a 1-year follow-up.

**Methods:** We administered the n-back task to 2897 healthy children aged 7–11 years old from 39 schools in Barcelona (Spain). The task consisted of 2 levels of complexity or loads (2- and 3-back) and 2 different stimuli (numbers and words). Participants performed the task four times from January 2012 to March 2013. To study the trajectories during the follow-up, we performed linear mixed-effects models including school, individual and age as random effects.

**Results:** We observed improvements related to age in n-back outcomes *d*′, HRT and accuracy, as well as reduced cognitive growth at older ages in *d*′ and HRT. Greater improvements in performance were observed at younger ages, in 2-back, in verbal rather than numerical stimuli and in girls compared to boys. Boys responded faster at baseline, while girls showed increased growth in 2-back numbers. Children with ADHD (Attention Deficit and Hyperactivity Disorder) symptoms (15% of boys and 6% of girls) had a lower working memory at baseline, but they showed similar cognitive growth trajectories in numbers variants of the task, as compared to children without ADHD symptoms. However, the age-related improvement in response speed was not observed in children with ADHD symptoms.

**Conclusions:** Changes in n-back outcomes reflected developmental trajectories in 1-year follow-up. The present results suggest that the repeated administration of this task can be used to study the factors that may alter the cognitive development during childhood.

## Introduction

Neuropsychological instruments are widely used in epidemiological studies to measure the impact of environmental factors on neurodevelopment. Most of the studies assess the neuropsychological status in one or several time points. However, individual trajectories of cognitive development have been rarely studied in epidemiological studies on neurodevelopment as it is usually performed in other health outcomes, such as lung function (Lødrup Carlsen et al., 2014), growth (Giles et al., 2015), and weight (Carling et al., 2015). The study of cognitive trajectories, based on several measures in short periods of time, would improve neurodevelopment characterization as a process in order to detect alterations in the growth pattern caused by social, environmental and other factors (Lei et al., 2015). The major criticism is the learning effect due to repeated administration of the same test in short periods (Dikmen et al., 1999).

Among the cognitive functions, working memory (WM) is highly related to learning ability and intelligence (Gathercole et al., 2003). WM emerges in early childhood and develops until adulthood (Cowan et al., 1999; Gathercole et al., 2003; Østby et al., 2011; Ullman et al., 2014). Tamnes et al. (2013) recently reported an annual increase in WM task scores of 5.4% during childhood, but this rate progressively diminishes during early (2.4%) and late adolescence (0.3%, although not statistically significant). N-back task is a widely used computerized test to measure WM (Nelson et al., 2000; Vuontela et al., 2003). Compared with other standard WM tasks, such as Reading Span Test (Daneman and Carpenter, 1980) or the operation span task (Turner and Engle, 1989), the responses that the participants must provide in n-back task are far simpler. In these traditional WM measures the subject has to perform a cognitive task while certain information has to be recalled later. In n-back task, individuals are not asked to recall any information but to recognize it. The n-back is a continuous recognition task in which participants must decide whether a stimulus was previously presented in certain conditions. The computerized format of this task provides increased objectivity that allows the use of highly precise outcomes, as well as efficiency, which allows to collect a big amount of data in a relatively short time. This task has been demonstrated to be a valid instrument in cross-sectional epidemiological studies (Forns et al., 2014). Age-related patterns of n-back task in children and adolescents have been also studied in a recent cross-sectional study (Pelegrina et al., 2015). In addition, this task, embedded in a wider neuropsychological battery named “Cogstate,” has been validated for measuring cognitive change in children through repeated administrations, since only weak measurement error or practice effect was observed (Mollica et al., 2005).

The developmental trajectories of n-back task can be modulated by gender and ADHD (Attention Deficit and Hyperactivity Disorder) symptoms. Although, previous literature indicated that boys and girls develop executive processes at similar rates (Becker et al., 1987; Welsh et al., 1991), other research have found male advantage in developmental trajectories for navigation and spatial tasks (Krikorian and Bartok, 1998; Grön et al., 2000) and female advantage in verbal tasks (Dorfberger et al., 2009). Regarding ADHD, longitudinal neuroimaging data indicate that children with ADHD follow a trajectory of cortical development that is delayed by 2–3 years relative to their typically-developing peers (Shaw et al., 2007). The executive functions are especially affected in these children (Sergeant et al., 2002; Semrud-Clikeman et al., 2008).

The simplicity, objectivity, efficiency and validity of n-back task allow the investigation of developmental trajectories in large-scale studies with the final aim of being applied in epidemiological research. Although the age-related patterns of n-back task performance in children and adolescents have been already studied with a cross-sectional design (Pelegrina et al., 2015), to our knowledge, there are no previous attempts to study the age-related trajectories of n-back during childhood with a longitudinal approach. Here we studied a sizeable sample of children (*N* = 2897) who were assessed four times during a period of 1 year with the n-back task. This design allowed us not only to study individual trajectories, but also to explore practice effects of this task, since we were able to compare the performance of children at the same age with and without practice. This study would represent an improvement due to a better characterization of cognitive development in order to identify its determinants at a population level. Thus, we aimed to study whether repeated administrations of n-back task detect developmental trajectories in schoolchildren during a 1-year follow-up. We explored the potential modulation that some key factors may exert over the developmental trajectories, namely, age, sex, and ADHD symptoms. Based on the literature, we expect that: (1) children will increase n-back scores and they will decrease latencies during the study period; (2) the oldest children will show less progression than the younger ones; (3) girls will show an advantage in verbal tasks and (4) children with ADHD symptoms will show a delayed developmental pattern.

## Materials and methods

### Participants

This study is part of the BREATHE (BRain dEvelopment and Air polluTion ultrafine particles in scHool childrEn) project, which aims to analyze the association between air pollution and cognitive development of schoolchildren. The BREATHE project was conducted from January 2012 to March 2013 in 36 schools of Barcelona, and 3 in Sant Cugat del Vallès, a smaller city near Barcelona (Catalonia, Spain). All the families of children attending these 39 schools in the 2nd, 3rd, and 4th primary grades (aged from 7 to 10 years [mean = 8.55, *SD* = 0.88]) were invited to participate via mail and/or project presentations in the schools. The total number of participants was 2904 (59%), but 7 of them were excluded from the analysis due to mental, motor or sensory impairment reported by the school. Fifty percent of the participants were males and 55% of mothers had a university degree. All parents and legal guardians signed the informed consent approved by the Ethical Committee of the IMIM-Parc Salut Mar.

### Instruments

#### Neuropsychological testing

Children were evaluated in groups of 10–20 every 3 months over four repeated sessions using the computerized n-back task. The duration of the sessions was 25 min. We followed a strict protocol in order to minimize measurement error. Firstly, the task was administered in a quiet and spacious room in the school. Secondly, children wore headphones to avoid noise disturbances. Thirdly, there was a trained examiner for every 3–4 children. Fourthly, sufficient distance between children reduced interaction among them. Fifthly, the test instructions were always explained following the same structure and by the same examiner. Finally, some variables such as day of the week, season, noise, weather, time of the day, quality of the session and incidences during the session were collected. These variables were included in the models in order to test their influence in the main results, but no significant effects were found (data not shown). Session date and child's grade (2nd, 3rd, and 4th primary grades) were also recorded in the sessions.

In the n-back task the subjects were required to monitor a series of stimuli presented in the center of the laptop's screen and they had to respond whenever a given stimulus is the same as the one presented *n* trials previously (1-, 2-, and 3-back). These different conditions are known as loads and in the highest cognitive load (i.e., 3-back) the demands on WM are stronger. The stimuli used in this study were numbers and words in black-color font. The difficulty of the stimuli presented was adapted to the development of the children to avoid the “ceiling effect” where there is concentration of most of the subjects in the maximum scores. Thus, numbers were 10 single digits for 2nd and 3rd grades (0–9), while participants in 4th grade had 10 double digits (21, 39, 47, 15, 62, 71, 83, 90, 50, and 68). The difficulty of the words was adapted for each grade. The following Catalan words were used for 2nd grade: germà (brother), avi (grandfather), ningú (nobody), braç (arm), dent (tooth), petó (kiss), cullera (spoon), abric (coat), gol (goal), and cop (hit). For 3rd grade, we used the following words: oncle (uncle), metge (doctor), cabell (hair), coll (neck), oli (oil), camisa (shirt), mirall (mirror), empenta (push), galleda (washbowl), and calaix (drawer). For 4th grade, the words were: cosí (cousin), nebot (nephew), fuster (carpenter), cella (eyebrow), colze (elbow), fruita (fruit), trena (braid), raspall (brush), ferida (wound), and llibreria (bookcase). Stimuli were presented in a fixed central location on a white background for a 1500-ms duration with a 1000-ms interstimulus interval. All participants were required to press a specific keyboard button when the target appeared in the screen. Participants completed three blocks (1-, 2-, and 3-back) for each stimulus. In the 1-back level, the target was any stimulus that matched the stimulus immediately preceding it. In the 2-back level, the target was any stimulus that matched the one presented two trials previously. In the 3-back level, the target was any stimulus identical to the one presented three trials previously. Each block consisted of 25 trials. The first three trials of each block were never targets and 33% of stimuli of the following trials were targets. After each block, a short break (5–20 s) was provided to allow participants some rest. Upon completion of each target, children heard a motivational recorded sample (“woo hoo!”) and a smiling face appeared at the top left of the screen.

Direct measures (hits, correct rejections, false alarms, and misses) and hit reaction time (HRT) were obtained for each trial. We calculated the overall accuracy including both hits and correct rejections, and d prime (*d*′) for each block separately. This outcome is derived from signal detection theory and allows the distinction of signal and noise. Measures of *d*′ were computed as follows: *d*′ = z(hit rate) − z(false alarm rate). A higher *d*′ indicated better detection, and thus, a more accurate performance (Deserno et al., 2012).

The task was created using the psychology experiment computer program E-Prime version 2.0 (Psychology Software Tools Inc.), and was performed on laptops with a standard 15″ screen.

#### Covariates

Socio-demographic data including childbirth date, sex, maternal education level (primary or low, secondary and university), origin from child and parents, linguistic context and home addresses were obtained from a questionnaire completed by parents during 2012. We calculated children's age for each session based on birth date and session date. A neighborhood socio-economic status vulnerability index (based on level of education, unemployment, and occupation at the census tract; Sunyer et al., 2015) was calculated at the home address. Teachers reported ADHD symptoms of each child using the ADHD Criteria of Diagnostic and Statistical Manual of Mental Disorders, fourth edition (ADHD-DSM-IV) list (American Psychiatric Association, 2002). ADHD-DSM–IV consists of a list of 18 symptoms categorized in two separate symptom groups. These are inattention (nine symptoms) and hyperactivity/impulsivity (nine symptoms). Each ADHD symptom is rated on a 4-point scale (0 never or rarely, 1 sometimes, 2 often, or 3 very often). We recoded the options 0 and 1 as 0 (symptom absent), and ratings of 2 and 3 as 1 (symptom present; Gomez, 2007). We used a categorical variable of ADHD clinical criteria with four categories, according to the presence of 6 or more symptoms of each subtype: (a) no ADHD; (b) ADHD-inattentive; (c) ADHD-hyperactive/impulsive; and (d) ADHD-combined.

### Statistical analyses

The medians of *d*′, HRT and accuracy of each load (2- and 3-back) and stimulus (numbers and words) were obtained at the 4 different sessions. We performed analyses of variance (ANOVA) to test differences between loads and stimuli statistically. We created the two categorical variables “memory load” and “stimulus” for these analyses. We studied the change in task performance at 4th session vs. 1st session on each age group including the interaction between session and the grade in the models.

Due to the hierarchical structure of the data (children embedded within schools and repeated measures collected on a child over time) we performed multilevel mixed-effects linear regression models for each outcome to study the developmental trajectories across sessions. 1-back trials were not included in the analyses because a ceiling effect was observed. We included school, individual and age as random effects, and age (linear and quadratic terms to capture the nonlinearity in the growth trajectories, if any) as fixed effects. The quadratic model with random intercepts and random slope for each child is shown below:

$$\begin{array}{l} Y_{sit}\;=\left(\beta_{0}\;+\;u_{0s}\;+\;s_{0i\left(s\right)}\right)\;+\;\left(\beta_{1}\;+\;s_{1i\left(s\right)}\right)\\ \;\times \;age_{sit}\;+\;\left(\beta_{2}\;+\;s_{2i\left(s\right)}\right)\;\times \;age_{sit^{2}}\;+\;\varepsilon_{sit} \end{array}$$

Where *Y*_*sit*_ is the n-back outcome for individual *i* within school *s* at session *t, t* = {1,2,3,4}, *u*_*s*_ are random effects at school level, *s*_*i*(*s*)_ are random effects associated with the individual *i* within school *s*, and ε_*sit*_ are the residuals.

First, random effects associated with age were tested using likelihood-ratio tests. Afterwards, we included the interaction between age and sex, and stratified models were presented if the growth pattern differed according to sex. Then, we tested interactions between age and ADHD symptoms, and the models were also stratified when the interactions were statistically significant. Fixed effects were tested using Wald tests. To visualize the shape of the growth function, we plotted the average predicted curve and two 95% confidence bands, one accounting only for the fixed effects and the other one adding the variation of the random effects. Statistical significance was set at *p* < 0.05 and *p* ≤ 0.1 for interaction. Statistical analyses were done using R (3.0.2; R Foundation for Statistical Computing) and Stata 12.1 (Stata Corporation, College Station, Texas).

## Results

Table 1 shows the number of participants in each session by age group and sex. The characteristics of the sample by age group are reported in Table 2. The median age in the first testing session was 7.6 years old in 2nd grade, 8.7 in 3rd grade and 9.7 in 4th grade. Children had a Spanish origin in 83% of the younger children and in 85% of children in the oldest group. Maternal education level was high in half of the sample for all age groups and the majority of children use Catalan in the family context (43–46%). ADHD symptoms were more present in the oldest group (12%). Inattentive type was observed in 4% of the girls and 8% of the boys in this study. Hyperactivity symptoms were reported for 0.5% girls and 3% boys, and combined ADHD symptoms were detected in 1% and 4% of the girls and boys respectively.

**Table 1:** **Number of participants in each session by age group and sex**.

**Age group**	**Sex**	**S1**	**S2**	**S3**	**S4**
2nd	Girl	503	496	474	454
	Boy	502	526	501	494
3rd	Girl	453	480	465	451
	Boy	510	499	498	474
4th	Girl	373	382	368	363
	Boy	355	365	350	321
		2696	2748	2656	2557

2nd, second grade at session 1 (7–8 years old); 3rd, third grade at session 1 (8–9 years old); 4th, fourth grade at session 1 (9–10 years old); S1, session 1; S2, session 2; S3, session 3; S4, session 4.

**Table 2:** **Sample characteristics by age group (*n* = 2897)**.

	**2nd grade (*n* = 1083)**	**3rd grade (*n* = 1036)**	**4th grade (*n* = 778)**
Age at session 1 (mean, SD)	7.6 (0.32)	8.7 (0.36)	9.7 (0.35)
Sex (% girls)	49.3	48.8	51.5
**MATERNAL EDUCATION (%)**			
Primary or low	14.5	10.1	10.9
Secondary	23.6	26.1	32.8
University	56.9	56.9	50.3
Missings	5.0	7.0	6.0
**SES Vulnerability Index (%)**			
1st tertile	35.8	37.0	37.8
2nd tertile	31.1	31.6	28.7
3rd tertile	31.6	30.0	32.7
Missings	1.5	1.5	0.9
**ORIGIN (%)**			
Spanish	82.7	83.4	85.2
Other	16.1	15.1	13.4
Missings	1.2	1.5	1.4
**LINGUISTIC CONTEXT (%)**			
Bilingual (Spanish and Catalan)	12.4	13.2	11.3
Catalan	46.3	43.4	46.3
Spanish	27.6	29.1	31.5
Other language	8.8	7.4	5.0
Missings	5.0	6.9	5.9
**ADHD SUBTYPES (%)**			
No ADHD	89.4	87.6	86.9
ADHD-inattentive	6.0	6.0	6.4
ADHD-hyperactive/impulsive	1.4	1.5	2.4
ADHD-combined	2.5	1.6	3.1
Missings	0.7	3.3	1.2

SES, socio-economic status; ADHD, Attention Deficit and Hyperactivity Disorder.

Comparing the loads, 2-back scores were higher than 3-back with both stimuli (*p* < 0.001). The scores obtained using numbers were higher compared to verbal stimuli (*p* < 0.001). However, in 3-back, the scores were higher using words (*p* < 0.001). HRT decreased by session in all stimuli and loads. Children were faster in 2-back than in 3-back (*p* < 0.001), and the responses were more delayed using words, mainly in 3-back (*p* < 0.001).

**Figure 1:**
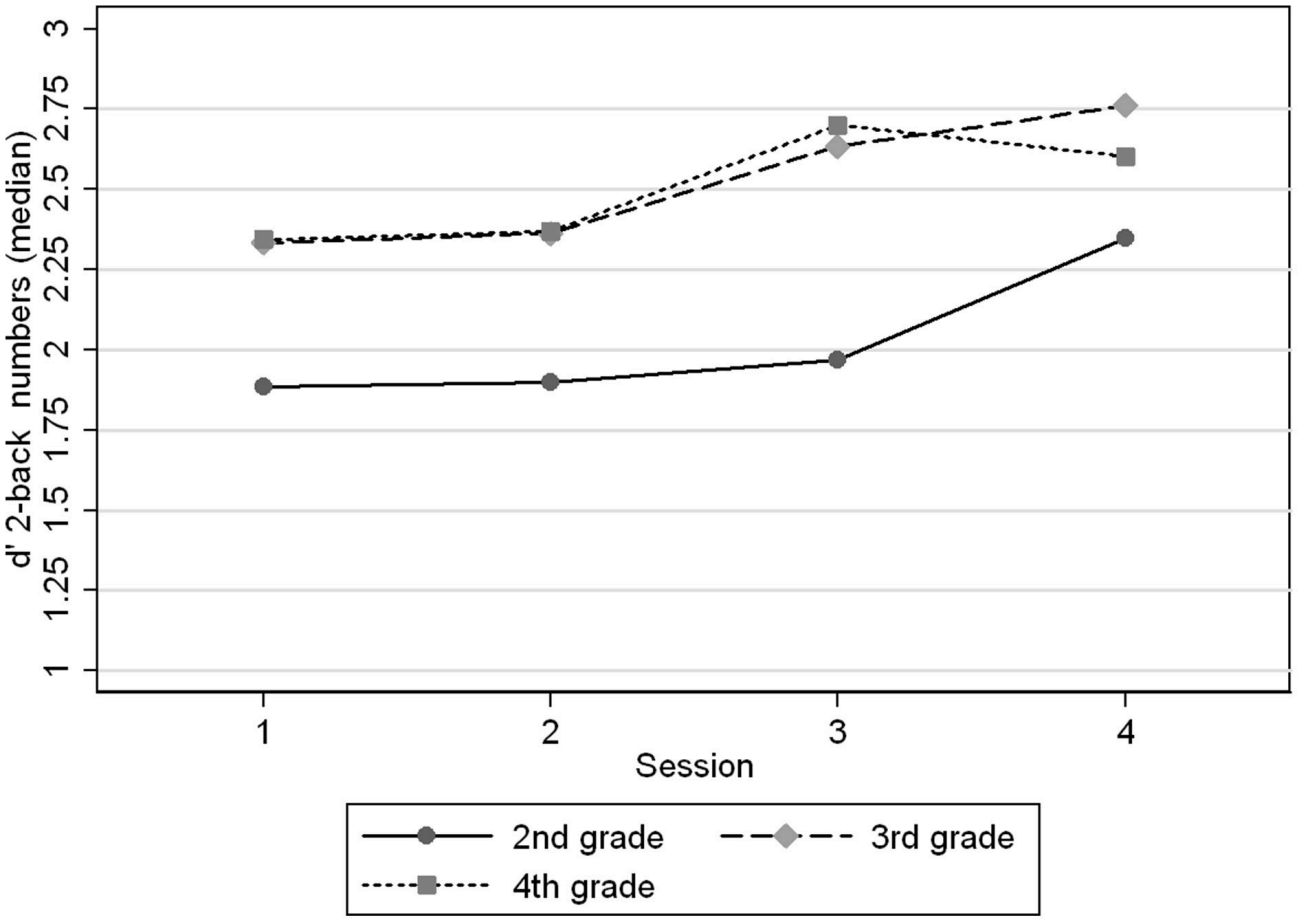
**Medians of *d*′ 2-back numbers by age group (grades) at different sessions**.

**Figure 2:**
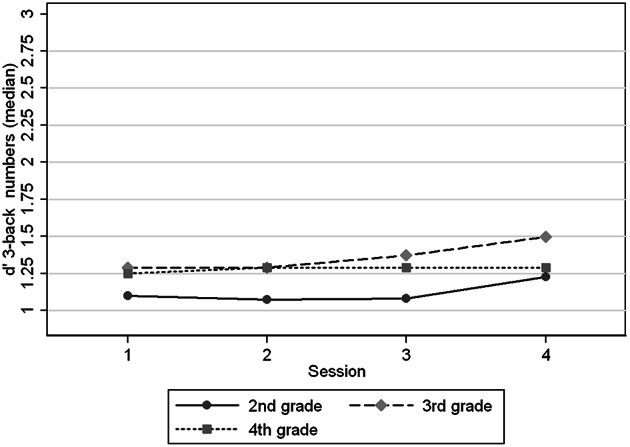
**Medians of *d*′ 3-back numbers by age group (grades) at different sessions**.

**Figure 3:**
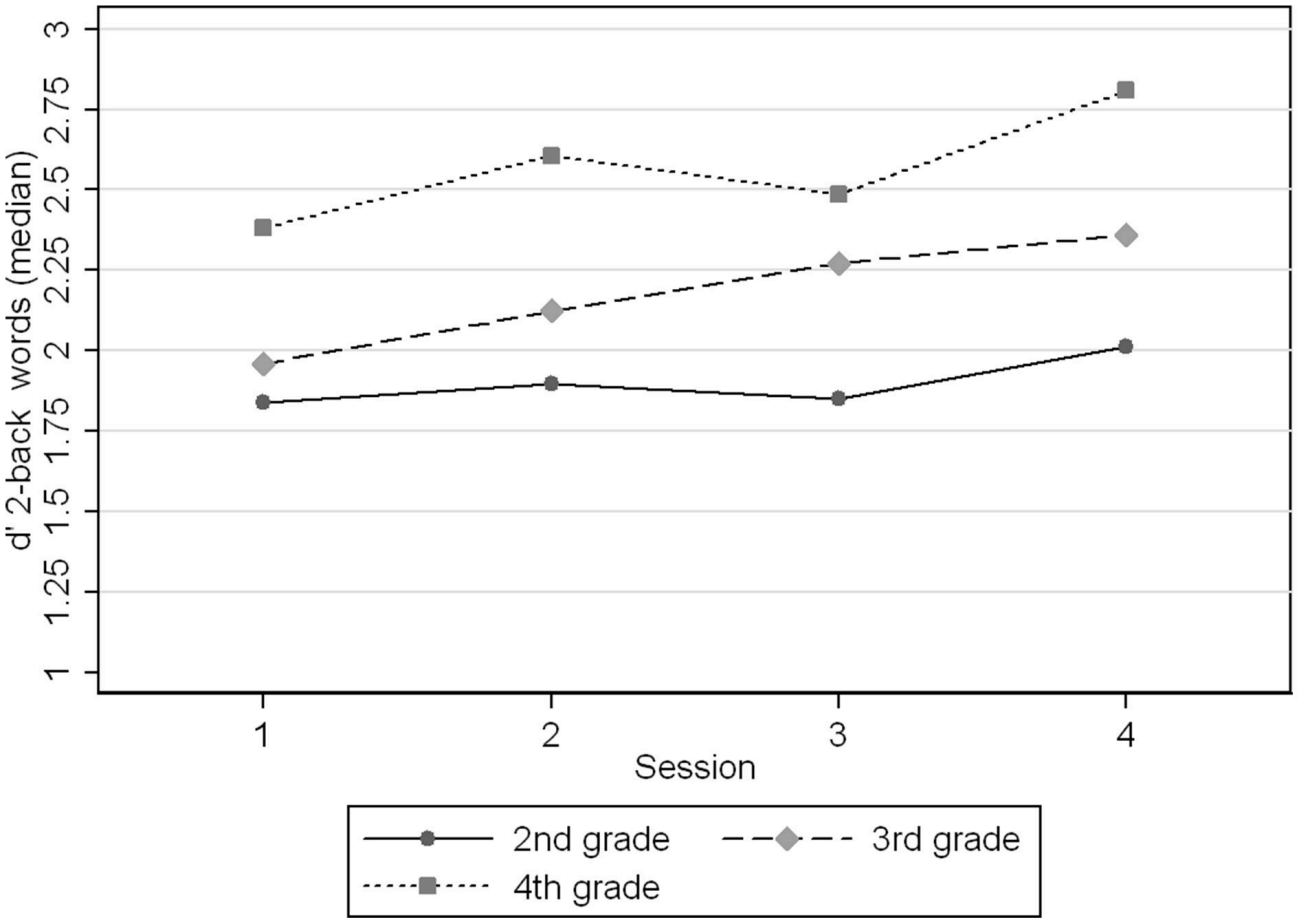
**Medians of *d*′ 2-back words by age group (grades) at different sessions**.

**Figure 4:**
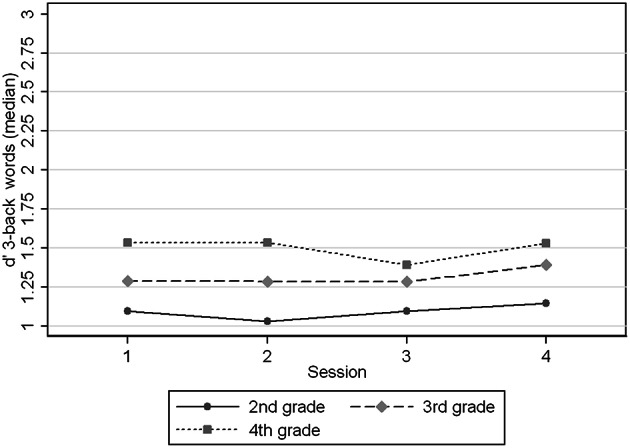
**Medians of *d*′ 3-back words by age group (grades) at different sessions**.

Figures 1–4 show the *d*′ medians of each task condition by session (1–4) and age group (2nd, 3rd, and 4th grade) to compare the performance in the task with and without practice at the same ages. We observed that children in 2nd grade reached the performance level of children in 3rd and 4th grades in 2-back numbers, while the two older groups performed at similar levels across the four sessions (Figure 1). We found significant interaction coefficients between session and age group in the 3rd (Coefficient: −0.20, *p* = 0.006) and 4th grades (Coefficient: −0.31, *p* < 0.0001) compared to the 2nd grade. In 2-back words the 3 age groups improved in a similar rate across the four visits, reaching the levels of the next age group in the 4th session. In 3-back conditions the levels in the 4th session did not reached the levels of the older groups in the 1st session. Children from 2nd and 3rd grades improved their performance between the 1st and 4th sessions, in contrast with the oldest children (4th grade). Significant interaction coefficients between session and grade were found in 3-back numbers (Coefficient: −0.11, *p* = 0.095) and words (Coefficient: −0.22, *p* = 0.001) in the 4th grade, being 2nd grade the reference group.

The medians of baseline scores by sex and ADHD symptoms are reported in Table 3. Boys performed better than girls in 3-back number trials (*p* < 0.05), and they were faster in all tasks. The groups of children with inattention and combined ADHD symptoms obtained the lowest *d*′ and accuracy scores (*p* < 0.0001) compared to children without ADHD symptoms. Children with inattention symptoms were also slower in 2-back numbers (*p* < 0.05), but they responded faster than children without ADHD symptoms in 3-back words (*p* < 0.05). Children with hyperactivity symptoms responded slower in 3-back numbers (*p* < 0.05).

**Table 3:** **Medians (p25, p75) of *d*′, hit reaction time (HRT, ms) and accuracy at baseline by sex and ADHD symptoms**.

**Outcome**	**Groups**	**2-back**		**3-back**	
		**Numbers**	**Words**	**Numbers**	**Words**
*d′*	Girls	1.98 (1.29, 3.27)	1.96 (1.29, 3.24)	1.12 (0.59, 1.90)	1.29 (0.64, 1.90)
	Boys	2.21 (1.31, 3.92)	1.98 (1.25, 3.49)	1.28 (0.51, 1.71)^*^	1.29 (0.60, 1.96)
	No ADHD	2.21 (1.39, 3.92)	1.98 (1.29, 3.43)	1.12 (0.59, 1.88)	1.29 (0.72, 1.90)
	ADHD-inattentive	1.53 (0.78, 2.34)^***^	1.43 (0.60, 2.41)^***^	0.80 (0.19, 1.53)^***^	1.03 (0.32, 1.53)^***^
	ADHD-hyperactive/impulsive	2.21 (1.62, 3.92)	2.38 (1.62, 3.92)	1.09 (0.78, 1.71)	1.52 (0.97, 2.12)
	ADHD-combined	1.74 (0.99, 2.80)^***^	1.89 (0.99, 2.63)^***^	1.03 (0.13, 1.29)^***^	1.03 (0.42, 2.12)^***^
HRT	Girls	766 (621, 938)	876 (732, 1022)	824 (659, 1006)	889 (741, 1042)
	Boys	729 (570, 882)^***^	829 (696, 979)^***^	799 (620, 960)^*^	868 (709, 1007)^*^
	No ADHD	746 (598, 908)	853 (713, 1002)	808 (641, 979)	882 (730, 1024)
	ADHD-inattentive	752 (612, 929)^*^	858 (669, 1024)	753 (639, 976)	853 (669, 994)^*^
	ADHD-hyperactive/impulsive	727 (598, 874)	835 (706, 927)	828 (598, 932)^*^	872 (745, 1000)
	ADHD-combined	793 (640, 968)	855 (697, 988)	811 (637, 946)	836 (698, 993)
Accuracy	Girls	88 (84, 96)	88 (80, 95)	80 (72, 88)	80 (72, 88)
	Boys	92 (84, 100)	88 (80, 96)	80 (72, 88)	80 (71, 88)
	No ADHD	92 (84, 100)	88 (80, 96)	80 (75, 88)	80 (72, 88)
	ADHD-inattentive	84 (76, 92)^***^	80 (68, 92)^***^	76 (68, 84)^***^	76 (64, 84)^***^
	ADHD-hyperactive/impulsive	89 (84, 100)	90 (80, 100)	80 (76, 88)	80 (72, 88)
	ADHD-combined	84 (76, 94)^***^	84 (68, 92)^***^	76 (67, 83)^***^	72 (60, 88)^***^

ADHD, Attention Deficit and Hyperactivity Disorder.

Wilcoxon rank-sum test was applied to compare boys and girls' performance and pairwise tests, adjusted for multiple comparisons using Tukey's honestly significant difference, to compare task performance between no ADHD (reference) and ADHD subtypes.

*** p < 0.0001;

* p < 0.05.

In the multilevel mixed-effects linear regression models strong age associations were found for all *d*′ outcomes. We observed quadratic curves with different degrees in all outcomes as well, indicating reduced cognitive growth at older ages (Table 4). Girls had an increased cognitive growth across the 1-year follow-up in comparison to boys, except for 2-back number trials, which trajectories were similar between boys and girls. Children with and without ADHD symptoms had the same growth pattern, except in 2-back word trials, in which children with combined ADHD symptoms had a more pronounced quadratic curve. That is, the age-related increases in performance diminished with age in both groups of children, however these reductions became more pronounced in children with ADHD symptoms. The inclusion of maternal education as a confounder did not change the results substantially.

**Table 4:** **Age-associated changes (coefficient, 95% CI)^†^ in the n-back outcomes during the 1-year follow-up**.

**Outcome**	**Age**	***p***	**Age^2^**	***p***
***d***′ **2-BACK NUMBERS**				
All	0.31 (0.26, 0.35)	<0.0001	−0.09 (−0.12, −0.07)	<0.0001
***d***′ **3-BACK NUMBERS**				
Girls	0.21 (0.16, 0.25)	<0.0001	−0.06 (−0.08, −0.03)	<0.0001
Boys	0.12 (0.07, 0.17)	<0.0001	−0.04 (−0.07, −0.01)	0.008
***d***′ **2-BACK WORDS**				
Girls	0.32 (0.27, 0.38)	<0.0001	−0.06 (−0.09, −0.02)	0.002
Boys	0.19 (0.14, 0.23)	<0.0001	–	
No ADHD	0.26 (0.22, 0.30)	<0.0001	−0.03 (−0.06, −0.002)	0.035
ADHD-combined	0.30 (0.06, 0.53)	0.014	−0.20 (−0.37, −0.04)	0.017
***d***′ **3-BACK WORDS**				
Girls	0.26 (0.21, 0.30)	<0.0001	−0.04 (−0.07, −0.01)	0.004
Boys	0.16 (0.11, 0.21)	<0.0001	−0.04 (−0.07, −0.01)	0.020
**HRT 2-BACK NUMBERS**				
Girls	−47.71 (−57.17, −38.25)	<0.0001	11.62 (5.88, 17.35)	<0.0001
Boys	−35.17 (−44.29, −26.05)	<0.0001	6.89 (0.93, 12.85)	0.023
**HRT 3-BACK NUMBERS**				
No ADHD	−19.75 (−27.78, −11.73)	<0.0001	6.83 (1.74, 11.91)	0.009
ADHD-inattentive	–		–	
**HRT 2-BACK WORDS**				
No ADHD	−52.68 (−59.79, −45.56)	<0.0001	8.35 (3.89, 12.81)	<0.0001
ADHD-combined	–		–	
**HRT 3-BACK WORDS**				
No ADHD	−28.07 (−35.93, −20.20)	<0.0001	7.01 (2.07, 11.95)	0.005
ADHD-hyperactive/impulsive	–		–	
**ACCURACY 2-BACK NUMBERS**				
No ADHD	0.56 (0.18, 0.93)	0.004	–	
ADHD-inattentive	1.71 (0.05, 3.36)	0.043	–	
**ACCURACY 3-BACK NUMBERS**				
Girls	–		–	
Boys	−0.89 (−1.48, −0.30)	0.003	–	
**ACCURACY 2-BACK WORDS**				
No ADHD	1.57 (1.13, 2.01)	<0.0001	–	
ADHD-combined	–		–	
**ACCURACY 3-BACK WORDS**				
Girls	1.42 (0.83, 2.00)	<0.0001	–	
Boys	–		–	
No ADHD	0.50 (−0.09, 1.09)	0.097	0.42 (0.06, 0.77)	0.020
ADHD-combined	–		–	

CI, Confidence Interval; d′, d prime; HRT, Hit Reaction Time, ADHD, Attention Deficit and Hyperactivity Disorder.

† Coefficients obtained from multilevel mixed-effects linear regression models including school, individual and age as random effects.

Stratified results by sex and ADHD are provided when p-value for interaction ≤ 0.1.

− No effect.

Regarding HRT, the same pattern as *d*′ was observed, children became faster with age, as well as the quadratic curves indicated reduced growth at older ages. We observed interactions between age and sex in the association with 2-back numbers, being the age effect more pronounced in girls. We also observed interactions between age and ADHD symptoms in the association with the other conditions. The stratified analyses showed that while the associations between age and the outcomes were strong in children with no ADHD symptoms, this association was not observed in children with ADHD symptoms.

We also observed interactions in the accuracy outcomes. Sex interacted with age in the association with 3-back numbers and words, while no age effect was found in girls using numbers stimuli, a negative effect of age was observed in boys. Contrarily, a positive age effect was found in girls using words, while no effect was found in boys. ADHD symptoms also interacted with age in the association with 2-back numbers and words and 3-back words. While the accuracy of 2-back numbers increased more with age in children with ADHD inattention symptoms, no age effect was observed in children with ADHD combined symptoms in 2- and 3-back words. Moreover, 3-back words in children without ADHD showed an increased cognitive growth pattern at older ages.

**Figure 5:**
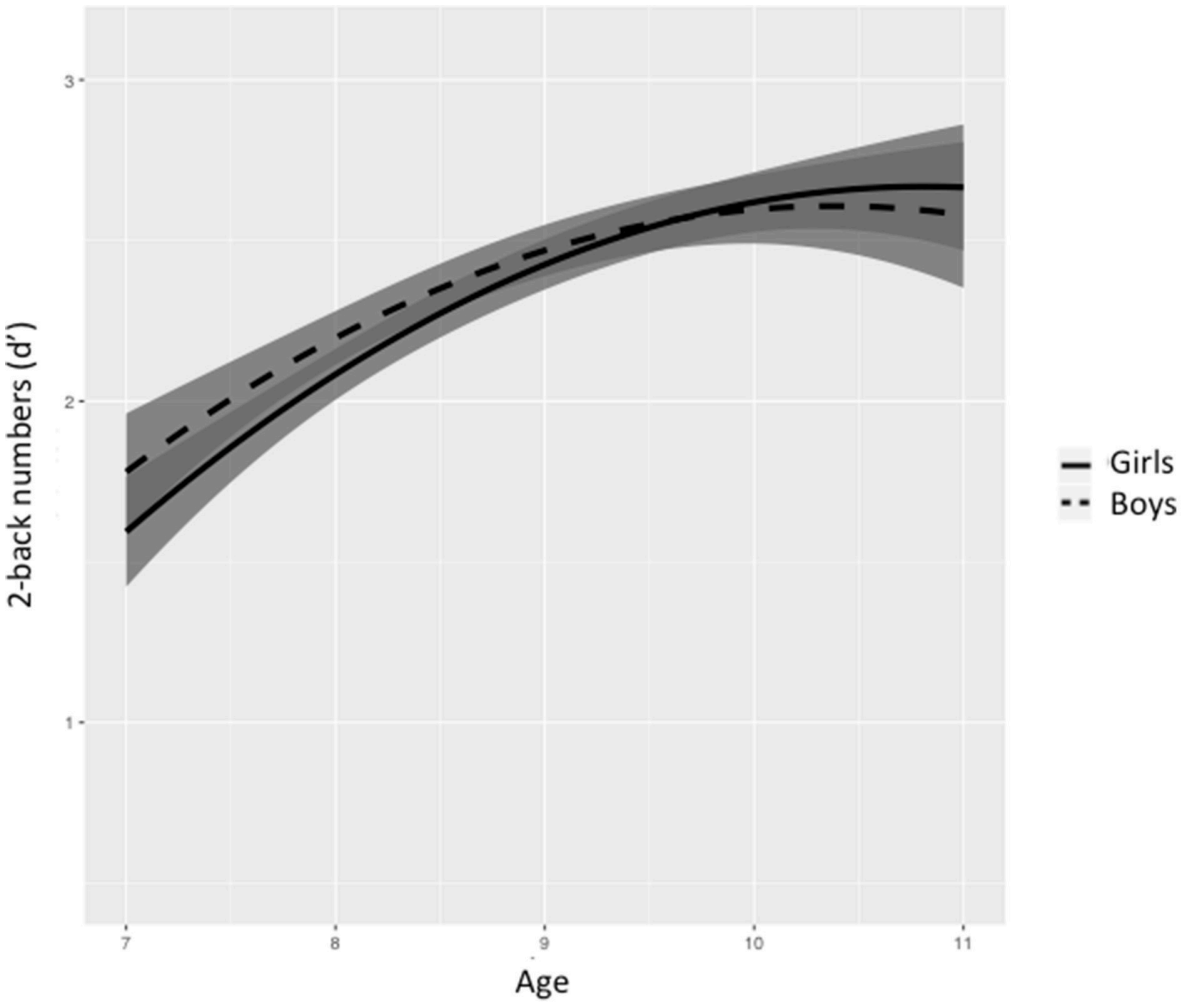
**Age-associated predicted curves for *d*′ 2-back numbers**. Average predicted curve and two 95% confidence bands, one accounting only for the fixed effects part and another one adding the variation of the random effects. Stratified results by sex are provided.

**Figure 6:**
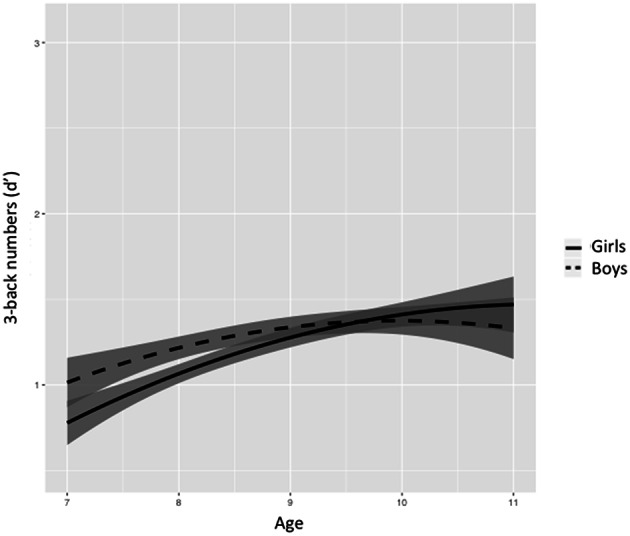
**Age-associated predicted curves for *d*′ 3-back numbers**. Average predicted curve and two 95% confidence bands, one accounting only for the fixed effects part and another one adding the variation of the random effects. Stratified results by sex are provided.

**Figure 7:**
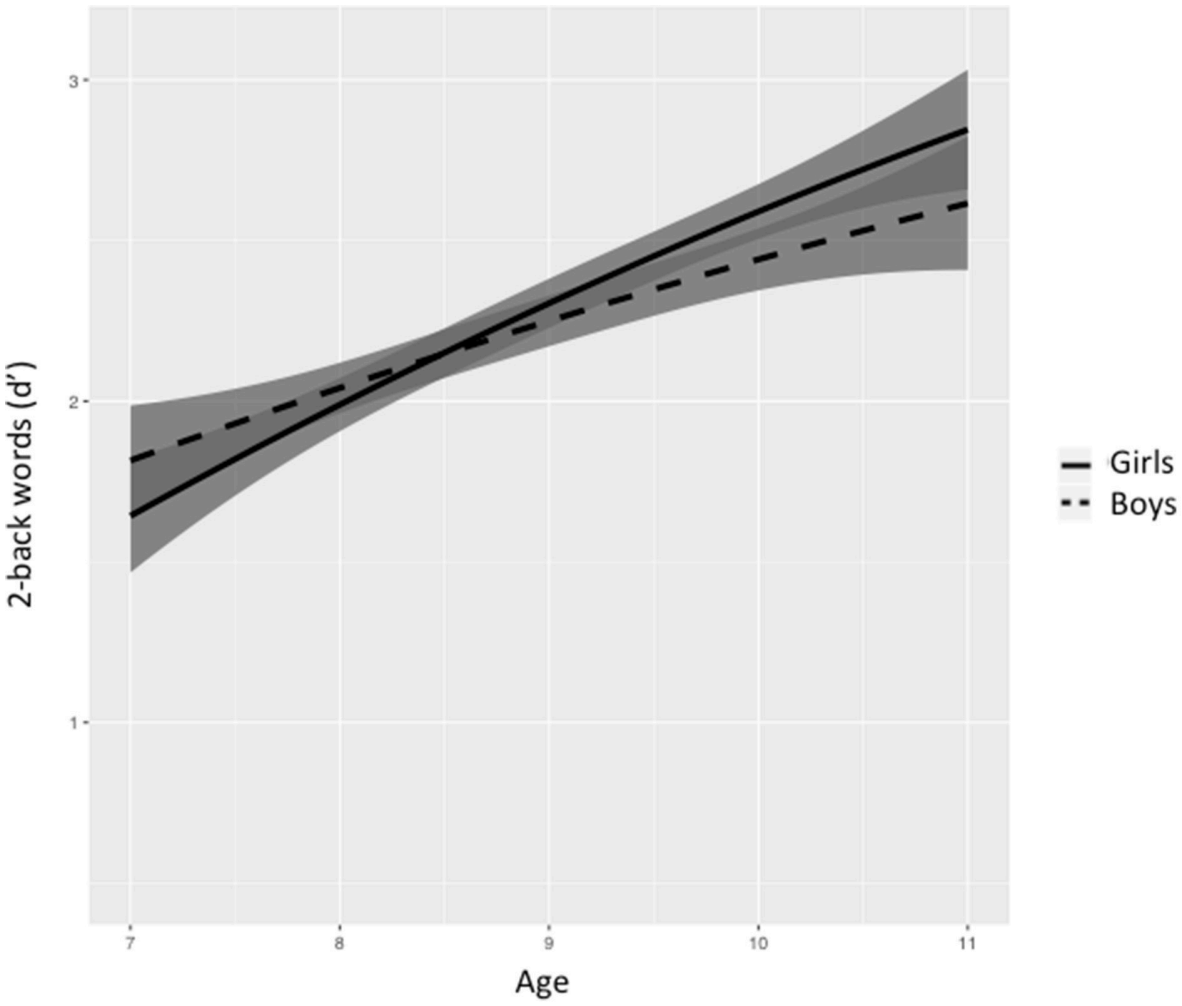
**Age-associated predicted curves for *d*′ 2-back words**. Average predicted curve and two 95% confidence bands, one accounting only for the fixed effects part and another one adding the variation of the random effects. Stratified results by sex are provided.

**Figure 8:**
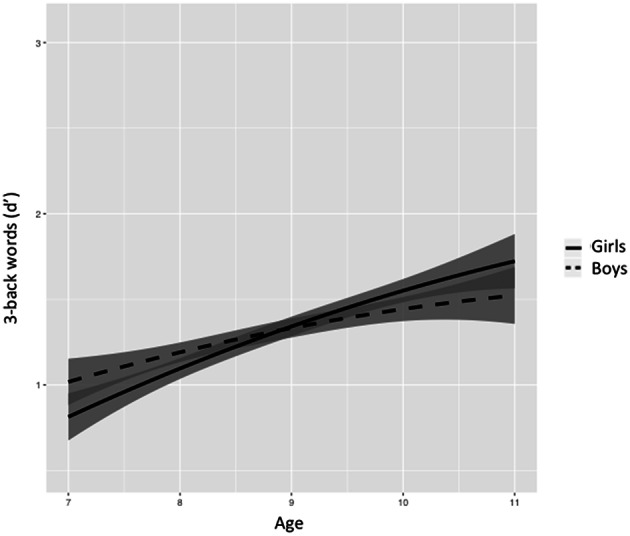
**Age-associated predicted curves for *d*′ 3-back words**. Average predicted curve and two 95% confidence bands, one accounting only for the fixed effects part and another one adding the variation of the random effects. Stratified results by sex are provided.

Figures 5–8 represent changes in *d*′ observed during 1 year period in 2- and 3-back performance using numbers and words stimuli. The curves showed a rapid improvement at younger ages and they stabilized at the end of the age range studied. Significant interactions between sex and age were found in 2-back words and 3-back numbers and words. Girls obtained lower scores at younger ages, but they showed a further improvement than boys across the 1-year period, resulting in higher scores at older ages and steeper slopes.

## Discussion

The current study has shown, for the first time, that n-back task outcomes were able to detect developmental trajectories in children from 7 to 11 years old from the general population in a period of 1 year. Specifically, we observed a rapid improvement in *d*′ score of n-back at younger ages, and more pronounced in 2-back than 3-back. This trend decreased at older ages. The cognitive growth measured with *d*′ was more pronounced in girls as compared to boys and similar in children with and without ADHD symptoms using numbers, although the baseline performance in children with ADHD symptoms was lower. Children without ADHD symptoms became faster in their responses with age, while no improvement were observed in children with symptoms. Boys responded faster at baseline, while girls showed increased growth in the HRT of 2-back numbers. The accuracy of the tasks increased with age, being this trend stable across ages and even more pronounced at older ages in 3-back words and in girls. This pattern was not observed in children with ADHD symptoms. In addition, we did not observe important differences in the median *d*′ scores of children at same ages in different sessions (i.e., 1st session vs. 4th session), indicating that our results were not due to practice.

The growing pattern related to age observed in n-back performance is consistent with previous studies of WM development (Ciesielski et al., 2006; Tamnes et al., 2013; Vuontela et al., 2013) and, specifically, with n-back age-related trends (Pelegrina et al., 2015). The literature described that WM experiences a high development during childhood (Ciesielski et al., 2006; Best and Miller, 2010; Vuontela et al., 2013). In the present study, we applied a repeated-measurement design and we found age-related trajectories within individuals. In line with Pelegrina's study (Pelegrina et al., 2015), the cognitive change was more pronounced at the youngest ages, which corresponds to a period of rapid growth in frontal lobe connections described between the ages of 7 and 9 years (Anderson, 2002; Vuontela et al., 2009). WM capacity increases during childhood (Halford et al., 1998) due to the establishment of executive networks, which contribute to the improvement of high load tasks, whereas the success in low load tasks depends on the activation of brain areas more related to short-term memory (Thomason et al., 2009). The developmental trajectories differ depending on the complexity of the task, with less demanding tasks being mastered earlier in development (Luciana et al., 2005; Conklin et al., 2007). Thus, the brain areas supporting processes related to higher demands on storage, processing, and executive control in 3-back may be still immature in the participants of this study (Best and Miller, 2010), which could explain the restricted improvement observed in high load tasks, compared to 2-back tasks.

Our results showed that verbal n-back improvement was slightly higher than numerical n-back. This finding could be due to different maturation rates, since previous studies have demonstrated that children perform better with numerical than verbal WM tasks (Luciana et al., 2005; Conklin et al., 2007). Moreover, the reading ability is still developing during the first years of primary school, which may explain the greater growth observed in this study (Yeatman et al., 2012). The semantic content of words could facilitate the use of effective strategies in maintaining information in mind, which could explain the higher *d*′ scores observed in verbal 3-back, compared to the numerical variant of the task (Shivde and Thompson-Schill, 2004; Rose, 2013), as well as the increased accuracy growth at older ages in our study.

Girls showed greater change over the age range studied although boys obtained higher scores at younger ages. These findings have been previously reported and could be due to earlier maturation peaks among girls (Vuontela et al., 2003; Pelegrina et al., 2015). Comparing the different stimuli, the performance in “numbers” trials was better among boys, while girls scored higher in “words” trials. Although, some studies did not observe differences in brain activation during WM tasks by sex (Schmidt et al., 2009), there is some evidence about males' advantage in abstract thinking (Lejbak et al., 2011) and better verbal skills in females (Torres et al., 2006). Furthermore, it has been suggested that females tend to use verbal strategies across all versions of the n-back task (Lejbak et al., 2011).

Previous studies have demonstrated that children with ADHD symptoms could have a later cognitive maturation (Shaw et al., 2011; Mous et al., 2014). In line with this evidence, our findings indicated that the performance in children with ADHD symptoms at baseline was below the scores of the children without ADHD symptoms, although in the numerical task the growing pattern was similar between them. We found a more pronounced slowing down of *d*′ score at older ages in 2-back words and the accuracy of n-back words did not show a growing pattern in this group of children. These results may suggest a different maturation pattern according to the stimuli in children with ADHD symptoms. Regarding this finding, these children may present a delay in the development of reading abilities as compared to children without symptoms (Willcutt and Pennington, 2000), which could interfere with the improvement in the task performance using verbal stimuli. The absence of a HRT growing pattern in children with ADHD symptoms may be explained by the high variability in response speed that has been observed in these children (López-Vicente et al., 2014).

Some methodological limitations should be noted. The order of the stimuli included in this study, which was numbers first and words second, was based on the difficulty of each task variant and was the same in all testing sessions. This non-randomization of stimuli presentation may imply a bias regarding attention levels, which could be higher to the first stimuli, which were numbers, or practice within session, which could benefit the last trials or words trials. Although, the inclusion of the three outcomes *d*′, HRT and accuracy strengthened the developmental trajectories measurement, HRT has some limitations that should be mentioned. First, it is a mean of response time of the correct hits of each trial, and therefore, it could be based only on one hit. Second, it may be altered by an impulsive response pattern, resulting in an overrated score. Some considerations should be mentioned regarding the practice effect of this task. The higher performance level obtained in numerical n-back task by 3rd grade children as compared to 4th grade at same age can be explained by the fact that the difficulty of the stimuli was adapted to the developmental level of the children. Thus, participants in 3rd grade had single digits, while 4th grade children had double digits. Moreover, these differences may also reflect practice effects; while children in the 4th grade performed the test for the first time, children in the 3rd grade had previously performed the test at the same age. This implies that, in this specific case, session or the learning of the task across the sessions would explain a part of the improvement observed in n-back performance. Assuming a weak practice effect, in line with previous literature (Mollica et al., 2005), what we consider cognitive growth may also include some learning of the task over the four testing sessions. The information about ADHD symptoms was reported by teachers, thus we lack important information about the occurrence of these symptoms in other settings, such as home (Amador-Campos et al., 2006; Dirks et al., 2011; Korsch and Petermann, 2014). Regarding the external validity, we have to bear in mind the participants' social status (55% had high maternal education level), and their generation, since digital devices are part of the daily life of these children and this could restrict the generalization of the results to other populations.

The strengths of our study are the large sample size and the longitudinal design using repeated measurements (four times) in 1-year follow-up. The short intervals between assessments provided more precision on the developmental trajectories. The inclusion of different age groups in the study at the same time allowed us to observe the child cognitive development in different critical periods of brain maturation and also to explore practice effects of the task. The load and stimuli variety of n-back task in this study allowed us to detect different developmental patterns, as different loads and stimuli processing involve different brain areas. A major strength of this study is its use of multilevel mixed-effects linear regression modeling, due to the presence of multiple sources of variability in the data (i.e., age, sex, and stimulus). Furthermore, the real-life setting increased the ecological validity of the study.

Overall, n-back task detected age-related trajectories in primary schoolchildren from the general population. In addition, this task showed different developmental patterns by sex and ADHD symptoms. The present results suggest that the repeated administration of this task can be used to study the factors that may alter the cognitive development during childhood.

## Author contributions

ML, JF, and JS conceptualized and designed the study and drafted the initial manuscript. ME, RG supported and supervised the statistical analyses and revised the manuscript. MA coordinated and supervised data collections and critically reviewed the manuscript. ES, JJ supervised the interpretation of the results and critically reviewed the manuscript. MB, NS designed the data collection instruments and critically reviewed the manuscript.

### Conflict of interest statement

The authors declare that the research was conducted in the absence of any commercial or financial relationships that could be construed as a potential conflict of interest.
